# Understanding the importance of key risk factors in predicting chronic bronchitic symptoms using a machine learning approach

**DOI:** 10.1186/s12874-019-0708-x

**Published:** 2019-03-29

**Authors:** Huiyu Deng, Robert Urman, Frank D. Gilliland, Sandrah P. Eckel

**Affiliations:** Department of Preventive Medicine, University of Southern California, 2001 N. Soto Street, MC-9234, Los Angeles, CA 90089 USA

**Keywords:** Bronchitic symptoms, Air pollution, Machine learning, Gradient boosting model, Prediction model

## Abstract

**Background:**

Chronic respiratory symptoms involving bronchitis, cough and phlegm in children are underappreciated but pose a significant public health burden. Efforts for prevention and management could be supported by an understanding of the relative importance of determinants, including environmental exposures. Thus, we aim to develop a prediction model for bronchitic symptoms.

**Methods:**

Schoolchildren from the population-based southern California Children’s Health Study were visited annually from 2003 to 2012. Bronchitic symptoms over the prior 12 months were assessed by questionnaire. A gradient boosting model was fit using groups of risk factors (including traffic/air pollution exposures) for all children and by asthma status. Training data consisted of one observation per participant in a random study year (for 50% of participants). Validation data consisted of: (1) a random (later) year in the same participants (*within*-participant); (2) a random year in participants excluded from the training data (*across*-participant).

**Results:**

At baseline, 13.2% of children had asthma and 18.1% reported bronchitic symptoms. Models performed similarly within- and across-participant. Previous year symptoms/medication use provided much of the predictive ability (across-participant area under the receiver operating characteristic curve (AUC): 0.76 vs 0.78 for all risk factors, in all participants). Traffic/air pollution exposures added modestly to prediction as did body mass index percentile, age and parent stress.

**Conclusions:**

Regardless of asthma status, previous symptoms were the most important predictors of current symptoms. Traffic/air pollution variables contribute modest predictive information, but impact large populations. Methods proposed here could be generalized to personalized exacerbation predictions in future longitudinal studies to support targeted prevention efforts.

**Electronic supplementary material:**

The online version of this article (10.1186/s12874-019-0708-x) contains supplementary material, which is available to authorized users.

## Background

Pediatric bronchitic symptoms, especially among children diagnosed with asthma, have been underappreciated and understudied [1–3] and pose a significant clinical and public health burden, with substantial clinical costs, loss of quality of life, and school absences [4–6]. Reliable prediction of chronic respiratory symptoms and an understanding of the relative importance of determinants would support prevention efforts, particularly amongst children with asthma who are at the greatest risk [6].

Previous studies have identified environmental and clinical risk factors for respiratory symptoms and exacerbations [1, 2, 6–9]. For example, ambient air pollution and traffic-related air pollution near busy roads (concentrations of traffic pollutants: particulate matter, black carbon, total nitrogen oxides (NOx), and nitrogen dioxide (NO_2_)) have been shown to be associated with asthma exacerbations and respiratory symptoms such as bronchitis and wheeze. Other risk factors include previous medical history, obesity, presence of allergens (e.g. cockroaches), and exposure to second hand smoking [6, 7]. The relative importance amongst the risk factors in predicting bronchitic symptoms has yet to be established.

There have been relatively few studies that have taken personalized approaches to predicting exacerbations or symptoms that incorporate demographic, environmental, and clinical risk factors. One such study focused on predicting asthma, wheeze, and eczema using a large heterogeneous set of attributes in a cross-sectional population setting [10]. Longitudinal information on predictors and exacerbations provide stronger causal evidence. In this study, we aimed to use longitudinal data from the Southern California Children’s Health Study (CHS) to predict annual assessments of chronic bronchitis symptoms using indoor exposures, ambient air pollution exposures and other susceptibility factors, and to evaluate the role of traffic/ambient air pollution in predicting the bronchitic symptoms.

## Methods

### Study participants

Participants were selected from the most recent CHS cohort followed from 2003 to 2012 in 13 Southern California communities. This cohort consists of schoolchildren recruited from kindergarten and first grade classrooms in 2003 (baseline year), at ages ~ 5 through 7 years. Baseline and annual follow-up questionnaires administered to parents (through 2008) and students (after 2008) collected information on potential risk factors and our outcome of interest: bronchitic symptoms over the past 12 months (hereafter referred to as BCP), which was defined as bronchitis, a daily cough for 3 months in a row, or congestion/phlegm other than when accompanied by a cold.

### Potential risk factors

We developed the following risk factor groupings:

#### Sociodemographic factors

The baseline questionnaires collected demographic information, including: age, gender, language of the study questionnaire (Spanish or English), race/ethnicity (Hispanic white, non-Hispanic white and other), child’s health insurance coverage, body mass index (BMI) percentile, and parent’s education level. Annual BMI percentile was calculated by applying to Center for Disease Control age- and sex-specific growth charts [11].

#### Indoor/home exposures

At baseline, information was collected on the ownership of any pets (including dogs and cats), housing conditions (presence of pests, carpet, mildew, water damage, and gas stove), and perceived parental stress. Second-hand tobacco smoke exposure in the home was based on the question “Does anyone living in this child’s home currently smoke cigarette, cigars or pipes on a daily basis inside the home?” that was included in the annual questionnaire.

#### Traffic/air pollution exposures

Outdoor concentrations of particulate matter of less than 2.5 μm in aerodynamic diameter (PM_2.5_, μg/m^3^) and 10 μm (PM_10_, μg/m^3^), nitrogen dioxide (NO_2_, ppb), and ozone (O_3_, ppb) were measured at central sites in each of the 13 communities. Community-specific annual averages of the 24-h PM_10_, PM_2.5_, nitrogen dioxide, and of the 10 am to 6 pm averages of ozone were calculated based on these air pollution monitoring stations.

Traffic-related pollution exposures were estimated using CALINE4 line-source dispersion model based estimates at the residence. CALINE4 freeway NOx and non-freeway NOx, estimated on an annual average, was selected as a surrogate for the complex mixture traffic-related pollution.

#### Symptoms/medication use

Annual questionnaires assessed the presence of: wheeze, rhinitis (“in the past 12 months, has your child had a problem with sneezing or a runny or blocked nose when he/she did not have a cold or the flu?”), itchy eyes (“…has this nose problem been accompanied by itchy/watery eyes”), and any asthma medication use over the prior 12 months. Medication use was assessed based on questions about any rescue, controller and other medication use for asthma or wheezing in the last 12 months. Photographic charts of medications and inhalers were used to collect information on use of specific medications.

#### Asthma/eczema

Baseline questionnaires recorded the ever presence of eczema, asthma status, age of first doctor diagnosis with asthma (if appropriate), and parents’ asthma status. At each study visit, asthma status was based on a yes/no response to the question “Has a doctor ever diagnosed this child as having asthma?”

Time-varying, annually assessed risk factors were lagged a study year to allow the previous risk factor value to predict bronchitic symptoms in the current year. When the risk factor was missing in the previous year, the previous value (i.e., two study years earlier) was used. Time-varying risk factors were: height, BMI percentile, exposure to secondhand tobacco smoke at home, ambient air pollutants and traffic related pollutants, all symptoms and medication use factors, and asthma status. All records for a child were excluded if that child had a missing baseline questionnaire; records from a particular year were excluded if the child had missing BCP status or missing questionnaires in the previous two consecutive years.

### Statistical analysis

To predict BCP as a function of the potential risk factors, we used gradient boosting models (hereafter referred to as GBM), as implemented in the gbm package in R with a Bernoulli distribution for the binary outcome [12]. GBM is a machine learning method that combines a series of simple tree-based models [13]. Since it is based on trees, GBM has the advantageous features of: (a) allowing for various levels of interactions by controlling the number of splits in each tree (e.g., interaction depth = 1 indicates a tree with 1 split (and no interactions), interaction depth = 2 indicates trees with two splits each) and (b) accounting for observations with missing data by using a surrogate split method [14]. Unless otherwise specified, models had a shrinkage rate of 0.01, at least 10 observations per node of each tree, a bagging fraction of 0.5, and a training fraction of 0.5. The initial model consists of 2000 trees. The interaction depth (between 1 to 4) and optimal number of trees for the final model was determined using 5-fold cross validation (CV).

**Fig. 1:**
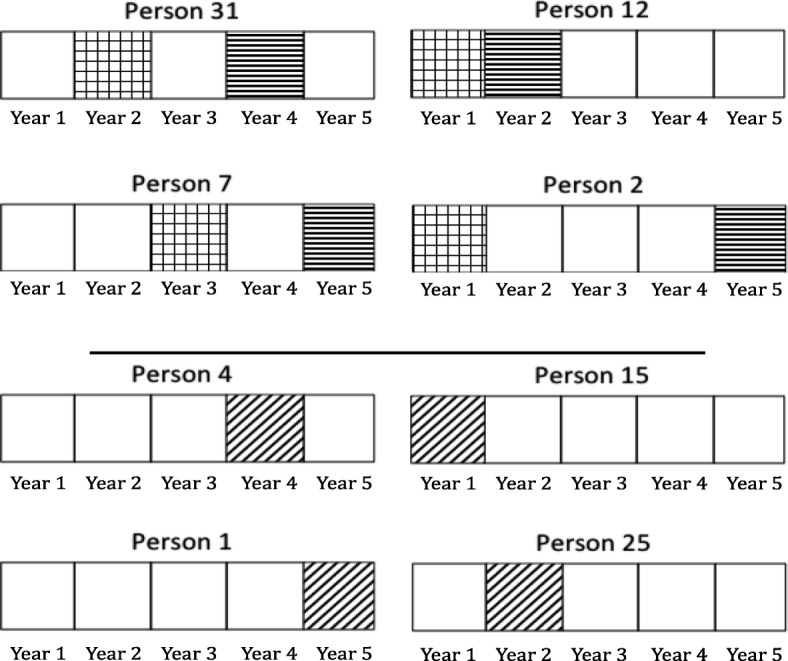
Conceptual division of the longitudinal, participant-level data into a training set and two tests sets (within- and across-participant). This figure illustrates the Conceptual division of the longitudinal, participant-level data into a training set and two tests sets (within- and across-participant). Suppose in hypothetical setting, data from 8 participants over 5 years were collected. Out of the 8 participants, a randomly selected 50% of study participants, two observations (at different study years) were randomly selected, which are person 31, 12, 7, and 2. Models were trained on the first of these observations (training set), denoted by: . Models were then validated using two complementary holdout test datasets. First, we considered the second (later) observation from the participants used to train the model (*within*-participant test set), denoted by . Then, we considered a random observation from the 50% of participants not included in the training set (*across*-participant test set), denoted by

We developed the following approach to train and validate our prediction models using the available longitudinal data, as illustrated in Fig. 1. For a randomly selected 50% of study participants, two observations (at different study years) were randomly selected. Models were trained on the first of these observations (training set), using 5-fold cross validation to tune model parameters. Models were then validated using two complementary holdout test datasets. First, we considered the second (later) observation from the participants used to train the model (*within*-participant test set). Second, we considered a random observation from the 50% of participants not included in the training set (*across*-participant test set).

Prediction models were constructed for all participants and for the subgroups consisting of children ever reporting asthma during the study period and those never reporting asthma. We performed subgroup analysis because: (1) children with asthma may have different key risk factors for BCP, (2) the impacts of a given risk factor may be different (e.g., increased) for children with asthma as compared to children without, (3) it may be easier (or harder) to predict BCP in children with asthma as compared to children without, and (4) potential differences in underreporting of symptoms between children with and without asthma may impact a prediction model trained simultaneously to both groups of children. Models were estimated using all potential risk factors and then, separately, using each of the five risk factor groups. Models for all participants included the variables: ever diagnosed with asthma and time varying asthma status (diagnosis of asthma over the prior 12 months) as additional potential risk factors. Models for children with asthma included age at first diagnosis as another potential risk factor. In sensitivity analyses, the subgroup of children without asthma was further subdivided into children ever reporting rhinitis and never reporting rhinitis.

We evaluated the predictive ability of a model using the area under the receiver operator characteristic curve (*AUC*) in the training data (using 5-fold CV) and in the two holdout test datasets. We also calculated the accuracy, sensitivity, and specificity for the within- and across- participant training sets at the optimal threshold on the predicted probability of bronchitic symptoms that jointly maximizes the distance to the identity (diagonal) lines in the cross-validated training data [15]. The accuracy, sensitivity, and specificity were calculated used the following formulas:

1 $$ \mathrm{Sensitivity}=\frac{{\mathrm{N}}_{\mathrm{TP}}}{{\mathrm{N}}_{\mathrm{TP}}+{\mathrm{N}}_{\mathrm{FN}}}, $$

2 $$ \mathrm{Specificity}=\frac{{\mathrm{N}}_{\mathrm{TN}}}{{\mathrm{N}}_{\mathrm{TN}}+{\mathrm{N}}_{\mathrm{FP}}}, $$

3 $$ \mathrm{Accuracy}=\frac{{\mathrm{N}}_{\mathrm{TP}}+{\mathrm{N}}_{\mathrm{TN}}}{{\mathrm{N}}_{\mathrm{TP}}+{\mathrm{N}}_{\mathrm{FN}}+{\mathrm{N}}_{\mathrm{TN}}+{\mathrm{N}}_{\mathrm{FP}}}, $$

where TP is true positive, TN is true negative, FP is false positive, and FN is false negative [16]. To ensure results robust to sampling variation, we retrained the GBMs and calculated the average AUCs and accuracies using 50 different random training sets and their corresponding holdout test datasets.

To better understand the predictive ability of specific risk factors in the “black box” GBM, we used relative influence, a statistic based on the number times a variable is involved in a split, weighted by the squared improvement of the model as a result of the split [17]. Higher relative influence values indicate that a variable has greater predictive ability [18]. For the models with all potential risk factors, we retrained the models to include only the top ten risk factors, based on relative influence. We then visualized the marginal associations of each of these 10 risk factors with the outcome using partial dependence plots. These plots display the effect of the given predictor on the outcome after marginalizing out all other predictors [17]. The correct way to marginalize out the other predictors is to numerically integrate them out over a grid of value of the given predictor, which can be computationally intensive. Thus, the common approach is to fix the other marginalized predictors to their sample mean and then calculated the effect of the given predictor [19]. We retrained the GBMs using 50 different random training sets and we presented the relative influence of the top ten risk factors (based on the median relative influence across the 50 training sets) and displayed partial dependence plots for GBM models from the first 5 of these random training sets.

Finally, we compared the performance of our GBM models with a classical logistic regression approach. For all participants and by asthma status, we developed logistic regression models that included the main effects of the top ten risk factors from the GBMs. Binary risk factors were included using the typical indicator variable approach and continuous risk factors were modeled using approaches motivated by the partial dependence plots (e.g., categorized). We retrained the logistic regression models using the 50 different random training sets and calculated the average AUCs in the corresponding holdout test datasets, and reported the model estimate using one of the random training sets.

All analyses were conducted in R version 3.3.2 (http://www.R-project.org).

## Results

The 4548 participants had information available from 2 to 7 visits each (average of 4.9). At baseline, participants were on average 6.5 years old, approximately half male (51.1%), and primarily Hispanic White (55.7%). 13.2% of the participants reported a diagnosis of asthma (Table 1). The baseline prevalence of bronchitic symptoms was 18.1% overall (36.7% in asthmatics and 11.5% in non-asthmatics). Of those children reporting BCP at baseline, 54% also reported BCP at the first follow-up year. Of those children not reporting BCP at baseline, 12.8% reporting BCP at the first follow-up year.

**Table 1:** Selected Characteristics of CHS participants with and without a lifetime history of physician diagnosed asthma at study entry^a^

Variable	All participants (*N* = 4548), Mean (SD) or N (%)	Asthma (*N* = 1199), Mean (SD) or N (%)	No asthma (*N* = 3349), Mean or (SD) (SD) or N (%)
Sociodemographic			
Age (Years)	6.5 (0.7)	6.5 (0.7)	6.5 (0.7)
Gender			
Male	2324 (51.1%)	695 (58.0%)	1629 (48.6%)
Female	2224 (48.9%)	504 (42.0%)	1720 (51.4%)
Spanish language questionnaire	1148 (25.2%)	207 (17.3%)	941 (28.1%)
Race/ethnicity			
Hispanic white	2531 (55.7%)	610 (50.9%)	1921 (57.4%)
Non-Hispanic white	1453 (32.0%)	402 (33.5%)	1051 (31.4%)
Other	564 (12.4%)	187 (15.6%)	377 (11.3%)
Insurance status	3813 (88.2%)	1057 (92.1%)	2756 (86.8%)
BMI percentile	61.1 (29.8)	63.6 (29.4)	60.2 (29.9)
Education level			
Less than 12th grade (did not finish high school)	942 (21.9%)	179 (15.6%)	763 (24.2%)
Completed grade 12 (high school)	824 (19.2%)	216 (18.8%)	608 (19.3%)
Some college or technical school	1628 (37.8%)	512 (44.6%)	1116 (35.4%)
Completed 4 years of college	501 (11.6%)	131 (11.4%)	370 (11.7%)
Some graduate training after college	408 (9.5%)	111 (9.7%)	297 (9.4%)
Indoor/home exposures			
Any pets at home	2387 (54.6%)	680 (58.9%)	1707 (53.1%)
Any pests at home	2811 (68.0%)	777 (70.4%)	2034 (67.0%)
Carpet at home	4030 (92.6%)	1079 (93.0%)	2951 (92.5%)
Mildew at home	1018 (24.8%)	323 (30.0%)	695 (22.9%)
Water damage at home	602 (14.0%)	185 (16.3%)	417 (13.2%)
Gas stove at home	3701 (85.5%)	989 (86.3%)	2712 (85.3%)
Parental stress^b^	4.1 (2.9)	4.1 (2.9)	4.0 (2.9)
Secondhand smoke exposure	328 (7.5%)	94 (8.1%)	234 (7.3%)
Traffic/air pollution			
24-h average: PM_10_ (μg/m^3^)	37.4 (12.3)	37.6 (11.7)	37.3 (12.5)
24-h average: PM_2.5_ (μg/m^3^)	17.6 (6.5)	18.1 (6.3)	17.5 (6.5)
24-h average: NO_2_ (ppb)	22.5 (8.2)	23.3 (8.1)	22.2 (8.2)
8-h (10 am-6 pm) average O_3_ (ppb)	43.6 (8.7)	44.2 (8.3)	43.4 (8.8)
CALINE4 freeway NO_x_ (ppb)	15.8 (22.4)	15.3 (18.7)	15.9 (23.6)
CALINE4 non-freeway NO_x_ (ppb)	5.6 (4.7)	5.5 (4.4)	5.6 (4.8)
Symptoms/medication use			
Wheeze symptom	641 (14.6%)	500 (43.2%)	141 (4.4%)
Rhinitis (i.e. sneeze/runny nose symptoms)	1458 (33.2%)	622 (53.9%)	836 (25.9%)
Itchy eyes symptoms	843 (19.3%)	370 (32.3%)	473 (14.7%)
Any asthma/wheeze medication use	693 (16.2%)	565 (48.8%)	128 (4.1%)
Bronchitis symptoms	781 (18.1%)	416 (36.7%)	365 (11.5%)
Asthma/eczema			
Parent history of asthma	1067 (24.7%)	478 (42.2%)	589 (18.5%)
Lifetime history of eczema	618 (14.6%)	246 (21.9%)	372 (12.0%)
Asthma status^c^	599 (13.2%)	599 (50.0%)	N/A
Age of asthma onset (years)	5.8 (4.3)	5.8 (4.3)	N/A

^a^The characteristics were taken at the study entry in 2003

^b^Levels of parental stress were assessed on baseline questionnaire via the four–item version of the Perceived Stress Scale (PSS), a composite stress score ranging from 0 to 16. Higher stress score indicates higher stress level

^c^Reported doctor-diagnosed asthma at baseline year

We constructed GBMs with an interaction depth of 1 because there was little evidence that more complex trees increased predictive ability (CV AUC was similar for GBMs with interaction depths from 1 to 4 and highest for interaction depth of 1, as shown in Additional file 1: Table S1). As shown in Table 2 and Additional file 1: Table S2, the set of symptoms/medication use risk factors yielded GBMs with predictive ability in terms of AUCs, accuracies, sensitivities and specificities nearly as high as that of the GBM using the set of all risk factors. The predictive ability of traffic/air pollution exposures was relatively poor. Specifically, the average CV AUCs for all participant models fitted using: all risk factors, symptoms/medication use, or traffic/air pollution exposures were: 0.77, 0.75, and 0.52, respectively. Average AUCs in the models stratified by asthma status (Table 2) were similar to the average AUCs obtained from fitting models using data on all participants and then validating the model by asthma status (Additional file 1: Table S3).

**Table 2:** Average area under the receiver operating characteristic curve (AUC) of models fit separately with groups of risk factors for all participants, asthmatics, and non-asthmatics, for 50 different random training sets and their corresponding holdout test datasets

	Risk factor groupings^a^	AUC: CV	AUC: Across- participants test set	AUC: Within- participants test set	Across-subject test set accuracy at the optimal threshold^c^
All participants	All predictors	0.77	0.78	0.75	0.74
	Sociodemographic	0.56	0.56	0.58	0.55
	Indoor/home exposures	0.54	0.55	0.56	0.60
	Traffic/Air pollution exposures	0.52	0.53	0.52	0.55
	Symptoms/medication use	0.75	0.76	0.73	0.75
	Asthma/eczema	0.68	0.69	0.67	0.71
	BCP (lag 1) only^b^		0.71	0.68	0.79
	BCP (lag 1) and traffic/air pollution exposures	0.71	0.70	0.68	0.79
	Top 10 risk factors	0.77	0.78	0.75	0.75
Asthmatics	All predictors	0.70	0.71	0.69	0.67
	Sociodemographic	0.52	0.55	0.54	0.52
	Indoor/home exposures	0.50	0.54	0.54	0.52
	Traffic/Air pollution exposures	0.49	0.51	0.52	0.51
	Symptoms/medication use	0.70	0.71	0.69	0.67
	Asthma/eczema	0.54	0.56	0.56	0.50
	BCP (lag 1) only^b^		0.68	0.67	0.68
	BCP (lag 1) and traffic/air pollution exposures	0.67	0.68	0.67	0.68
	Top 10 risk factors	0.70	0.71	0.68	0.67
Non-Asthmatics	All predictors	0.71	0.71	0.70	0.76
	Sociodemographic	0.54	0.55	0.56	0.49
	Indoor/home exposures	0.52	0.54	0.56	0.51
	Traffic/Air pollution exposures	0.51	0.52	0.51	0.57
	Symptoms/medication use	0.69	0.70	0.68	0.77
	Asthma/eczema	0.55	0.57	0.57	0.71
	BCP (lag 1) only^b^		0.67	0.64	0.81
	BCP (lag 1) and traffic/air pollution exposures	0.67	0.66	0.64	0.84
	Top 10 risk factors	0.71	0.72	0.69	0.75

^a^Variables in each risk factor grouping are listed in the text

^b^Cross validation was not able to apply to the GBM models with 1 predictor variable. Thus, CV AUC and optimal number of tree based on cross validation were not produced. The total number of 2000 trees was used in the GBM models with 1 predictor variable

^c^The optimal threshold was determined by using the predicted probabilities from the cross-validation set

**Fig. 2:**
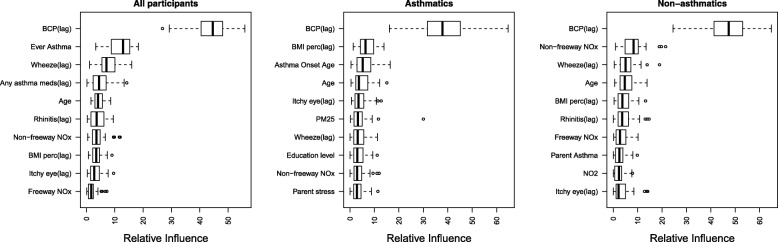
Boxplot of relative influence, for 50 different random training sets, of the top 10 risk factors in models fit using all predictor variables for all participants, asthmatics, and non-asthmatics

**Fig. 3:**
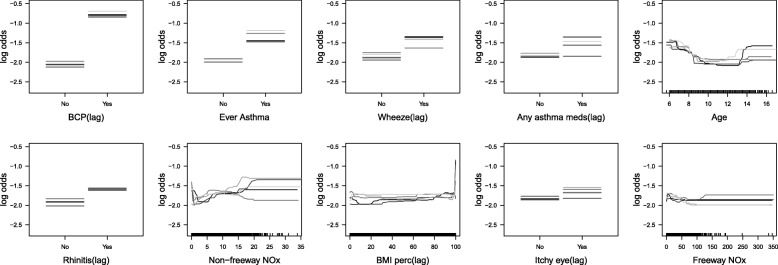
Partial dependence plots, for 5 different training sets, of the top 10 risk factors in models fit using only these 10 variables, for all participants. The distributions of the continuous predictors are demarcated using ticks above the x-axis

**Fig. 4:**
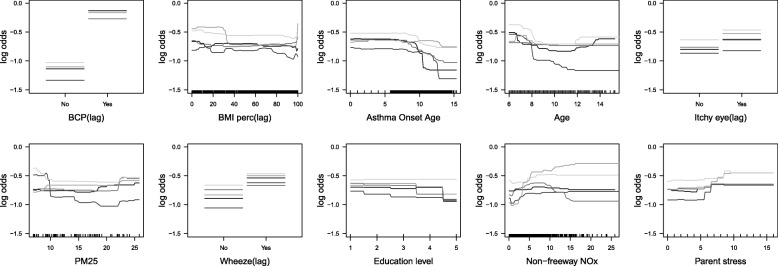
Partial dependence plots, for 5 different training sets, of the top 10 risk factors in models fit using only these 10 variables, for children with asthma. The distributions of the continuous predictors are demarcated using ticks above the x-axis

**Fig. 5:**
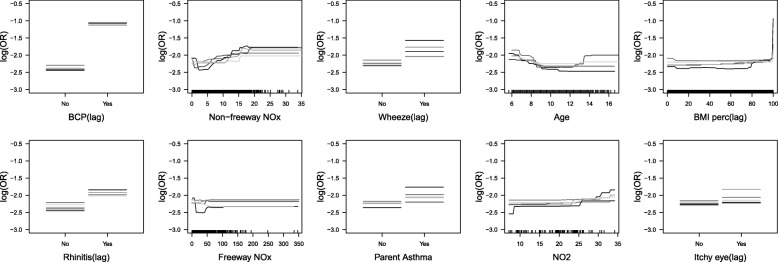
Partial dependence plots, for 5 different training sets, of the top 10 risk factors in models fit using only these 10 variables, for children without asthma. The distributions of the continuous predictors are demarcated using ticks above the x-axis

Relative influence analysis showed that having BCP in the previous year was the single most important predictor of current year BCP, overall, and within each asthma group (Fig. 2). Note that GBMs fit using only lag BCP had AUCs that were slightly lower than the GBMs with all predictors, implying that other predictors had modest predictive ability (Table 2). The average AUCs from models fit with the top 10 predictors were very similar to the average AUCs from models fit with all predictors (Table 2 and Additional file 1: Table S2). Partial dependence plots (Figs. 3, 4 and 5) indicated that, regardless of their asthma status, children with BCP in the previous year were more likely to have BCP in the current year. The top ten risk factors in both asthmatics and non-asthmatics also included: BMI percentile, itchy eyes, wheeze symptoms, age, and traffic/air pollution predictors (e.g., CALINE4 non-freeway NOx). The partial dependence plots suggested that children previously reporting wheeze were at increased risk of current BCP. Children (< 8 years old) and older children (> 14 years old) were also at increased risk. Non-freeway NOx had a positive association with BCP in all and non-asthmatic children. The directions of association were less clear with freeway NOx, BMI percentile, parent stress, and PM_2.5_. The results from the same analyses applied to the non-asthmatic subgroups showed no difference results from the non-asthmatic group (Additional file 1: Table S4 and Figure S1).

Based on the partial dependence plots for all participants, and by asthma status, all continuous predictors were categorized except freeway NOx and education level for the logistic regression analysis. Specifically, age was categorized as 0–8, 8–14, and 14+ years old; BMI percentiles were categorized as 0–95 and 95+; non-freeway NOx was categorized as 0–7, 7–15, and 15+ ppb; age of asthma onset was categorized at 0–9 and 9+; PM_2.5_ was categorized as 0–10, 10–20 and 20+ μg/m^3^; parental stress was categorized at 0–7 and 7+; NO2 was categorized as 0–10, 10–25, and 25+ ppb. Freeway NOx and education level was included as a continuous variable.

Logistic regression models had similar average test set AUC to GBM models with the same top 10 predictors (Additional file 1: Table S5, Figure S2, and Figure S3). Of the 10 predictors in the logistic regression model for all participants, asthmatics, and non-asthmatics (all of which were continuous), the maximum pairwise Pearson’s correlations were any asthma medication use and wheeze (R = 0.67), age of asthma onset and wheeze (R = -0.39), and itchy eyes and rhinitis (R = 0.68), respectively. Regression coefficients from the logistic regression models using one of the random training sets (Additional file 1: Table S6) should be interpreted with caution, since they are from prediction models that did not focus on adjustment for potential confounders and the covariates included in the models built for all children, and for children with and without asthma were different. That said, BCP in the previous year was significantly associated with the current year BCP (OR: 3.48 and 95% CI: 2.64–4.58 for all participants; OR: 3.01 and 95% CI: 2.03–4.49 for asthmatics; OR: 3.95 and 95% CI: 2.69–5.79 for non-asthmatics, with different adjustments for each model). Wheeze symptoms in the previous year were positively associated with current year BCP (OR: 1.60 and 95% CI: 1.13–2.26 for all participants; OR: 1.78 and 95% CI: 1.18–2.69 for asthmatics; OR: 1.93 and 95% CI: 1.12–3.28 for non-asthmatics, with different adjustments for each model). Itchy eye symptoms in the previous year had positive associations with current year BCP in the non-asthmatic subset (OR: 1.64 and 95% CI: 1.02–2.65). Children living in areas with high levels of non-freeway NOx [> 15 ppb] were at increased risk of BCP as compared to those with low levels [< 7 ppb] for all participants (OR: 2.51 and 95% CI: 1.36–4.47), and in the non-asthmatic subset (OR: 2.26 and 95% CI: 1.04–4.63).

## Discussion

We used gradient boosting models to build a prediction model and identify key risk factors for bronchitic symptoms in a large cohort of southern California schoolchildren. The best models had moderate discriminative performance (0.71 < AUC < 0.77), which might be considered good for our questionnaire-based outcome [20]. In general, previous symptoms—particularly previous year bronchitic symptoms—were the most informative predictors. Age, BMI percentile, itchy eye symptoms, parent stress, and traffic/air pollution risk factors (CALINE4 non-freeway NOx, and PM_2.5_) were amongst the top predictors in models fit by asthma status, and contributed modest additional predictive information. No indoor/home exposures were found to be predictive. The predictive performance of our models was similar in the within-subject and across-subject test sets which suggests, somewhat surprisingly, that these prediction models would perform similarly if applied to future observations in these study participants or to data from new participants.

Our findings on the importance of previous symptoms align with several previous studies of risk factors for chronic cough and asthma exacerbations in children. Forno and Celedon reviewed publications predicting asthma exacerbations in children and reported that a history of recent severe asthma exacerbation was a strong risk factor for subsequent exacerbations, regardless of disease severity or use of controller medication [6, 21–23]. In a 48-week Pediatric Asthma Controller Trial study with children age 6 to 14 years with middle-to-moderate persistent asthma, a history of an asthma exacerbation requiring a systemic corticosteroid in the past year was associated with a subsequent exacerbation (OR = 2.1, *p* < 0.001) [22]. Another study using administrative claims data from PharMetrics/IMS Health also confirmed the associations between suboptimal asthma control and history of recent asthma exacerbations with subsequent disease exacerbations [24].

Our finding that the top ten predictors included traffic-related pollution (CALINE4 freeway and non-freeway NOx) and regional, ambient PM_2.5_ is in line with previous reports that exposure to poor air quality is associated with bronchitic symptoms in children, but put these results in context since air pollution effects are smaller than effects of previous symptoms. Chen et al. reviewed the findings from the CHS and reported that children with physician-diagnosed asthma were at higher risk of developing chronic lower respiratory tract symptoms such as bronchitis and phlegm (BCP) if they lived in more polluted communities [1, 2]. Specifically, yearly questionnaire based bronchitic symptoms assessment from 1996 to 1999 were associated with the yearly variability of particulate matter with aerodynamic diameter less than 2.5 μg (1.09 μg/m^3^, CI: 1.01–1.17), NO_2_ (1.07 ppb^,^ CI: 1.02–1.13), and ozone (1.06 ppb, CI: 1.00–1.12) among the cohort of children with asthma in 12 Southern California communities. In a school-based, cross-sectional study in the San Francisco Bay Area in 2001, traffic-related pollution was associated with respiratory symptoms in children. Among those living at their current residence for at least 1 year, the adjusted odds ratio for bronchitis in relationship to an interquartile difference in NOx was 1.07 (95% CI, 1.00–1.14) [9]. Another CHS paper also indicates the relationship between new-onset asthma with traffic-related pollutions near homes and schools [25]. For example, asthma risk had a positive association with modeled traffic-related pollution exposure from roadways near homes (HR 1.51, 95% CI 1.25–1.82) and near schools (HR 1.45, 95% CI 1.06–1.98).

A recent study used several Bayesian network classifiers to predict the risk of asthma exacerbation in 65 pediatric asthma patients ages 1–14.5, with 2–4 measurements each [16, 26]. Using Backward Sequential Elimination and Joining algorithm (BSEJ), the authors achieved 93.84% accuracy and 90.9% sensitivity [16, 26]. The predictive performance of this model was better than we observed with the models in our dataset. Key differences include that our study was population-based whereas Spyroglou et al. recruited children from an asthma clinic who had recent cessation of asthma medication use. The prevalence of asthma exacerbation in the Spyroglou et al. study was 14.9%, similar to the prevalence in our study of BCP for all participants and non-asthmatics (18.1 and 11.5%, respectively), and lower than the prevalence of BCP amongst asthmatics (36.7%). Spyroglou et al. used only a within-participant test set to validate their models (held out the last observation for each participant) whereas we created within- and between- test sets to validate our models. Some features of the BSEJ algorithm make it less applicable to our study (e.g., only categorical predictors are permitted and it is challenging to apply to larger datasets or datasets with missing data).

We used gradient boosting model (GBM) to build prediction models for bronchitic symptoms. As GBM is comprised of multiple trees, successively constructed to overweight data that are hard to classify, it overcomes the biggest drawback of single tree models: their relatively poor predictive performance. Advantages of GBM include that it handles different types of predictor variables, it has a reasonable approach for highly correlated predictors (assigns importance to one of them, rather than splitting the importance across the highly correlated variables), is invariant to monotone transformations of individual predictor variables, is less sensitive to outliers, accounts for missing data using surrogate splits, and allows for automated detection of (potentially high-order) interactions, nonlinear relations [12–14, 27–29]. For example, for all participants, the association between non-freeway NOx and log odds of the current BCP was positive. According to the partial dependence plots, the association was nonlinear with a drastic increase when non-freeway NOx was greater than 15 ppb (Fig. 3). The logistic regression with the categorized non-freeway NOx also confirmed the nonlinearity – children living in areas with high levels of non-freeway NOx [> 15 ppb] were at higher risk of BCP as compared to those with low levels [< 7 ppb] for all participants (OR: 2.51, 95% CI: 1.36–4.47) and the risks of BCP were lower for children living in areas with medium levels of non-freeway NOx [> 7 ppb and < 15 ppb] (OR: 1.45, 95% CI: 1.10–1.91) (Additional file 1: Table S6).

A disadvantage of GBM (and prediction-driven machine learning modeling approaches in general) is that they are constructed under the goal of prediction. Effect estimates from these models (e.g., our logistic regression based on GBM results) should be interpreted with caution since the models were not constructed to account for potential confounders. We used relative influence and partial dependence plots to attempt to understand associations of key predictors. There are some drawbacks of these approaches. Specifically, one-way partial dependence plots assume no interaction effects [30]. In our case it was reasonable to consider only one-way partial dependence plots since our GBM models only had an interaction depth of 1. To ensure results were robust to sampling variation, we retrained the models using 50 multiple random training sets and evaluated the models using the corresponding test sets. Finally, we followed up on our GBM results by constructing logistic regression models that we found to have similar predictive performance. The logistic regression models can be readily applied to new study populations and clearly quantify the associations of individual predictors with the outcome.

This study applied machine learning to a longitudinal dataset, with up to 7 assessments per participant. Tree-based ensemble methods like random forest and GBM usually do not consider the dependency structure seen in longitudinal data [28]. If the dependency structure is ignored, correlation in the bootstrap samples used to produce each tree will lead to higher than expected correlation between trees and worse predictive performance. Relatively few studies have addressed the issue of how to model repeated measures data using tree-based models. Adler et al. considered data from a glaucoma registry with repeated measurements the left and/or right eyes of subjects and investigated the impact of varying the number of observations per subject in the training data for each individual base classifier (i.e., each tree) [28]. Specifically, Adler et al. compared the training data selection strategies consisting of: (1) one random observation per subject from a bootstrapped sample of subjects, (2) all observations per subject from a bootstrapped sample of subjects, (3) “naïve” bootstrap sample that ignored the correlation structure, (4) bootstrap samples of the subject-specific mean across all repeated measurements on a subject and (5) the newest observation of one selected eye per subject. Their results showed that sampling one observation was better than sampling all observations of each subject for both random forest and bagging classifiers. Our study took a similar approach and sampled one observation per participant for the ensemble-level training data. We further expanded on the Adler et al. method by creating complimentary within and across-participant holdout test datasets focusing on the generalizability of the model to future measurements on the same participants and completely new participants.

There are limitations to our study. In terms of the statistical methodology, to address the issue of correlation in longitudinal data, we used only up to two observations per participant for training and testing the prediction models and did not take full advantage of the up to 7 years of data per participant. Had the model been trained with repeated measures on each participant, we speculate that the within-participant holdout test AUC would have been higher. Future work might consider model-based longitudinal machine learning approaches such as RE-EM tree [31] for continuous outcome or a tree-based method using GEE (generalized estimating equations) for binary outcomes [32]. More generally, another limitation is that our outcome was assessed by annual self-reported questionnaire, so BCP symptoms might be underreported. Underreporting of symptoms would lead error in the outcome variable of our prediction models that is potentially systematic (e.g., different rates of underreporting for children who are asthmatic vs. non-asthmatic). Additional error in the outcome variable has the potential to harm the predictive ability of our models. However, if underreporting is consistent across years (i.e., a participant’s symptoms are underreported each year) the impacts of this underreporting on our prediction model may be attenuated since our models include reported symptoms in the previous year. Also, we developed models stratified by asthma status, which might attenuate any impacts of differential underreporting by asthma status.

In addition, our study did not consider some key individual level predictors such as individual genetic information, GxE exposures, diet, and indoor air. The lack of those predictors may explain why within-participants and between-participant predictions yielded similar results. This study also has a number of strengths. This study contains a large population-based sample of school children across several southern Californian communities with a wide range of risk factors including medical history, traffic and regional air pollutant exposures, and home exposures. Risk factors were investigated simultaneously to examine their relative importance in predicting the bronchitic symptoms. In addition, our study applied prediction modeling with all participants, and by asthma status.

## Conclusions

Our study applied gradient boosting models to predict bronchitic symptoms among school-aged children in a longitudinal framework, offering a novel approach to better understand predictive factors of this outcome. We found that children with previous bronchitic symptoms were at the highest risk of developing subsequent symptoms, while several traffic and regional air pollution exposures also contribute to the overall model predictive ability. A similar approach can be used in future panel studies with more highly time resolved data to create personalized prediction models to potentially predict and prevent acute asthma exacerbations or chronic reparatory disease.

## Additional file

Additional file 1: **Table S1.** Comparison of gradient boosting models fit for all participants and all predictors, for 50 different random training sets. **Table S2.** Accuracy, sensitivity, and specificity of models fit separately with groups of risk factors for all participants, asthmatics, and non-asthmatics, for 50 different random holdout test datasets. **Table S3**. Average AUC of models trained on various groups of risk factors using data from all participants and validated separately by asthma status, for 50 random training sets. **Table S4.** Average area under the receiver operating characteristic curve (AUC), accuracy, sensitivity, and specificity of models fit separately with groups of risk factors for non-asthmatics, non-asthmatics (rhinitis), and non-asthmatics (no rhinitis), for 50 different across- and within- participants holdout test datasets. **Table S5.** Comparison of gradient boosting models vs. logistic regression for all participants, asthmatics, and non-asthmatics averaged across 50 training sets. **Table S6.** Logistic regression results for all participants, asthmatics, and non-asthmatics for a random training set. **Figure S1.** Boxplot of relative influence, for 50 different random training sets, of the top 10 risk factors in models fit using all predictor variables for non-asthmatics, non-asthmatics (rhinitis), and non-asthmatics (no rhinitis). **Figure S2.** Area under the receiver operating characteristic curve (AUC) of the gradient boosting models and logistic regression model models fit separately with all risk factors and top 10 most important risk factors for 50 different random across-participant holdout test datasets. **Figure S3.** Area under the receiver operating characteristic curve (AUC) of the gradient boosting models and logistic regression models fit separately with all risk factors and top 10 most important risk factors for 50 different random within-participant holdout test datasets. (DOCX 3711 kb)

## Abbreviations

AUC: Area under the receiver operator characteristic curve
BCP: Bronchitis, cough, and phlegm
BMI: Body mass index
CALINE4: 4th generation Gaussian line source air qualify model developed by Caltrans
CHS: Children’s Health Study
CV: Cross validation
GBM: Gradient boosting model
GEE: Generalized estimating eqs.
HR: Hazard ratio
NO_2_: Nitrogen dioxide
NO_x_: Total nitrogen oxides
O_3_: Ozone
OR: Odds ratio
PM_10_: Particulate matter of less than 10 μm in aerodynamic diameter
PM_2.5_: Particulate matter of less than 2.5 μm in aerodynamic diameter
RE-EM tree: Random effects/EM tree
