# Supplementary material for: Understanding the importance of key risk factors in predicting chronic bronchitic symptoms using a machine learning approach
Source: BMC Med Res Methodol. 2019 Mar 29;19:70. doi: 10.1186/s12874-019-0708-x (PMC6441159; doi:10.1186/s12874-019-0708-x)
Supplement: Supplementary file 1 — Table S1. Comparison of gradient boosting models fit for all participants and all predictors, for 50 different random training sets. Table S2. Accuracy, sensitivity, and specificity of models fit separately with groups of risk factors for all participants, asthmatics, and non-asthmatics, for 50 different random holdout test datasets. Table S3. Average AUC of models trained on various groups of risk factors using data from all participants and validated separately by asthma status, for 50 random training sets. Table S4. Average area under the receiver operating characteristic curve (AUC), accuracy, sensitivity, and specificity of models fit separately with groups of risk factors for non-asthmatics, non-asthmatics (rhinitis), and non-asthmatics (no rhinitis), for 50 different across- and within- participants holdout test datasets. Table S5. Comparison of gradient boosting models vs. logistic regression for all participants, asthmatics, and non-asthmatics averaged across 50 training sets. Table S6. Logistic regression results for all participants, asthmatics, and non-asthmatics for a random training set. Figure S1. Boxplot of relative influence, for 50 different random training sets, of the top 10 risk factors in models fit using all predictor variables for non-asthmatics, non-asthmatics (rhinitis), and non-asthmatics (no rhinitis). Figure S2. Area under the receiver operating characteristic curve (AUC) of the gradient boosting models and logistic regression model models fit separately with all risk factors and top 10 most important risk factors for 50 different random across-participant holdout test datasets. Figure S3. Area under the receiver operating characteristic curve (AUC) of the gradient boosting models and logistic regression models fit separately with all risk factors and top 10 most important risk factors for 50 different random within-participant holdout test datasets. (DOCX 3711 kb) [file 12874_2019_708_MOESM1_ESM.docx]

**ONLINE SUPPLEMENT**

**Title:** Understanding the importance of key risk factors in predicting chronic bronchitic symptoms using a machine learning approach

**Running title:**

Predicting chronic bronchitic symptoms using machine learning

**Authors:** Huiyu Deng, Robert Urman, Frank D Gilliland, and Sandrah P Eckel

Table S1. Comparison of gradient boosting models fit for all participants and all predictors, for 50 different random training sets.

Table S2. Accuracy, sensitivity, and specificity of models fit separately with groups of risk factors for all participants, asthmatics, and non-asthmatics, for 50 different random holdout test datasets.

Table S3. Average AUC of models trained on various groups of risk factors using data from all participants and validated separately by asthma status, for 50 random training sets.

Table S4. Average area under the receiver operating characteristic curve (AUC), accuracy, sensitivity, and specificity of models fit separately with groups of risk factors for non-asthmatics, non-asthmatics (rhinitis), and non-asthmatics (no rhinitis), for 50 different across- and within- participants holdout test datasets.

Table S5. Comparison of gradient boosting models vs. logistic regression for all participants, asthmatics, and non-asthmatics averaged across 50 training sets.

Table S6. Logistic regression results for all participants, asthmatics, and non-asthmatics for a random training set.

Figure S1. Boxplot of relative influence, for 50 different random training sets, of the top 10 risk factors in models fit using all predictor variables for non-asthmatics, non-asthmatics (rhinitis), and non-asthmatics (no rhinitis).

Figure S2. Area under the receiver operating characteristic curve (AUC) of the gradient boosting models and logistic regression model models fit separately with all risk factors and top 10 most important risk factors for 50 different random across-participant holdout test datasets.

Figure S3. Area under the receiver operating characteristic curve (AUC) of the gradient boosting models and logistic regression models fit separately with all risk factors and top 10 most important risk factors for 50 different random within-participant holdout test datasets.

Table S1. Comparison of gradient boosting models fit for all participants and all predictors, for 50 different random training sets.

| Interaction depth | Average CV AUC^a^ | Average number of trees^b^ |
| --- | --- | --- |
| 1 | 0.7745 | 692.94 |
| 2 | 0.7732 | 487.34 |
| 3 | 0.7735 | 430.26 |
| 4 | 0.7719 | 392.08 |

^a^ The averaged CV AUC across 50 random samples.

^b^ The averaged number of trees, chosen via cross validation, across the 50 random samples.

Table S2. Accuracy, sensitivity, and specificity ^a^ of models fit separately with groups of risk factors for all participants, asthmatics, and non-asthmatics, for 50 different random holdout test datasets.

|  | Risk factor groupings | Accuracy: Across- participants test set | Sensitivity: Across- participants test set | Specificity: Across- participants test set | Accuracy: Within - participants test set | Sensitivity: Within - participants test set | Specificity: Within - participants test set |
| --- | --- | --- | --- | --- | --- | --- | --- |
| All participants | All predictors | 0.74 | 0.75 | 0.68 | 0.73 | 0.75 | 0.63 |
|  | Sociodemographic | 0.55 | 0.56 | 0.52 | 0.61 | 0.65 | 0.44 |
|  | Indoor/home exposures | 0.60 | 0.60 | 0.47 | 0.60 | 0.62 | 0.46 |
|  | Traffic/Air pollution exposures | 0.55 | 0.68 | 0.34 | 0.63 | 0.77 | 0.25 |
|  | Symptoms/medication use | 0.75 | 0.75 | 0.66 | 0.74 | 0.72 | 0.65 |
|  | Asthma/eczema | 0.71 | 0.71 | 0.60 | 0.70 | 0.72 | 0.57 |
|  | BCP (lag 1) only | 0.79 | 0.80 | 0.59 | 0.78 | 0.84 | 0.52 |
|  | BCP (lag 1) and traffic/air pollution exposures | 0.79 | 0.85 | 0.54 | 0.78 | 0.84 | 0.52 |
|  | Top 10 risk factors | 0.75 | 0.75 | 0.68 | 0.72 | 0.75 | 0.62 |
| Asthmatics | All predictors | 0.67 | 0.68 | 0.66 | 0.66 | 0.68 | 0.63 |
|  | Sociodemographic | 0.52 | 0.66 | 0.39 | 0.54 | 0.74 | 0.30 |
|  | Indoor/home exposures | 0.52 | 0.67 | 0.37 | 0.52 | 0.68 | 0.36 |
|  | Traffic/Air pollution exposures | 0.51 | 0.67 | 0.34 | 0.53 | 0.71 | 0.32 |
|  | Symptoms/medication use | 0.67 | 0.68 | 0.65 | 0.66 | 0.69 | 0.63 |
|  | Asthma/eczema | 0.50 | 0.69 | 0.38 | 0.48 | 0.46 | 0.59 |
|  | BCP (lag 1) only | 0.68 | 0.67 | 0.66 | 0.67 | 0.68 | 0.64 |
|  | BCP (lag 1) and traffic/air pollution exposures | 0.68 | 0.70 | 0.65 | 0.67 | 0.68 | 0.65 |
|  | Top 10 risk factors | 0.67 | 0.68 | 0.65 | 0.66 | 0.67 | 0.63 |
| Non-Asthmatics | All predictors | 0.76 | 0.74 | 0.58 | 0.75 | 0.74 | 0.55 |
|  | Sociodemographic | 0.49 | 0.63 | 0.42 | 0.58 | 0.66 | 0.41 |
|  | Indoor/home exposures | 0.51 | 0.70 | 0.34 | 0.51 | 0.72 | 0.34 |
|  | Traffic/Air pollution exposures | 0.57 | 0.68 | 0.35 | 0.65 | 0.77 | 0.24 |
|  | Symptoms/medication use | 0.77 | 0.76 | 0.55 | 0.76 | 0.73 | 0.56 |
|  | Asthma/eczema | 0.71 | 0.68 | 0.45 | 0.71 | 0.73 | 0.39 |
|  | BCP (lag 1) only | 0.81 | 0.80 | 0.51 | 0.82 | 0.84 | 0.43 |
|  | BCP (lag 1) and traffic/air pollution exposures | 0.84 | 0.89 | 0.42 | 0.83 | 0.88 | 0.39 |
|  | Top 10 risk factors | 0.75 | 0.74 | 0.59 | 0.73 | 0.71 | 0.57 |

^a^ The accuracy, sensitivity, and specificity were calculated based on the optimal threshold that was determined by using the predicted probabilities from the cross-validation sets.

Table S3. Average AUC of models trained on various groups of risk factors using data from all participants and validated separately by asthma status, for 50 random training sets.

| Categories of predictors | AUC: Across-participant test set | | AUC: Within-participant test set | |
| --- | --- | --- | --- | --- |
|  | Asthmatics | Non-asthmatics | Asthmatics | Non-asthmatics |
| All predictors | 0.71 | 0.73 | 0.70 | 0.70 |
| Sociodemographic | 0.54 | 0.55 | 0.55 | 0.58 |
| Indoor/home exposures | 0.54 | 0.55 | 0.54 | 0.57 |
| Traffic/Air pollution exposures | 0.51 | 0.53 | 0.51 | 0.52 |
| Symptoms/medication use | 0.71 | 0.72 | 0.69 | 0.68 |
| Asthma/eczema | 0.54 | 0.55 | 0.55 | 0.57 |

Table S4. Average area under the receiver operating characteristic curve (AUC), accuracy, sensitivity, and specificity ^a^ of models fit separately with groups of risk factors for non-asthmatics, non-asthmatics (rhinitis), and non-asthmatics (no rhinitis), for 50 different across- and within- participants holdout test datasets.

|  | Risk factor groupings^a^ | AUC: CV | AUC:  Across- participants  test set | Accuracy: Across- participants test set | Sensitivity: Across- participants test set | Specificity: Across- participants test set | AUC:  Within - participants  test set | Accuracy: Within - participants test set | Sensitivity: Within - participants test set | Specificity: Within - participants test set |
| --- | --- | --- | --- | --- | --- | --- | --- | --- | --- | --- |
| Non-Asthmatics | All predictors | 0.71 | 0.71 | 0.76 | 0.74 | 0.58 | 0.70 | 0.75 | 0.74 | 0.55 |
|  | Sociodemographic | 0.54 | 0.55 | 0.49 | 0.63 | 0.42 | 0.56 | 0.58 | 0.66 | 0.41 |
|  | Indoor/home exposures | 0.52 | 0.54 | 0.51 | 0.70 | 0.34 | 0.56 | 0.51 | 0.72 | 0.34 |
|  | Traffic/Air pollution exposures | 0.51 | 0.52 | 0.57 | 0.68 | 0.35 | 0.51 | 0.65 | 0.77 | 0.24 |
|  | Symptoms/medication use | 0.69 | 0.70 | 0.77 | 0.76 | 0.55 | 0.68 | 0.76 | 0.73 | 0.56 |
|  | Asthma/eczema | 0.55 | 0.57 | 0.71 | 0.68 | 0.45 | 0.57 | 0.71 | 0.73 | 0.39 |
|  | BCP (lag 1) only^b^ |  | 0.67 | 0.81 | 0.80 | 0.51 | 0.64 | 0.82 | 0.84 | 0.43 |
|  | BCP (lag 1) and traffic/air pollution exposures | 0.67 | 0.66 | 0.84 | 0.89 | 0.42 | 0.64 | 0.83 | 0.88 | 0.39 |
|  | Top 10 risk factors | 0.71 | 0.72 | 0.75 | 0.74 | 0.59 | 0.69 | 0.73 | 0.71 | 0.57 |
| Non-Asthmatics (Rhinitis) | All predictors | 0.71 | 0.71 | 0.77 | 0.75 | 0.57 | 0.69 | 0.75 | 0.73 | 0.56 |
|  | Sociodemographic | 0.54 | 0.55 | 0.47 | 0.66 | 0.39 | 0.56 | 0.56 | 0.74 | 0.32 |
|  | Indoor/home exposures | 0.53 | 0.53 | 0.58 | 0.69 | 0.34 | 0.56 | 0.58 | 0.69 | 0.37 |
|  | Traffic/Air pollution exposures | 0.52 | 0.53 | 0.59 | 0.68 | 0.35 | 0.51 | 0.67 | 0.77 | 0.24 |
|  | Symptoms/medication use | 0.68 | 0.69 | 0.78 | 0.77 | 0.55 | 0.67 | 0.77 | 0.72 | 0.56 |
|  | Asthma/eczema | 0.54 | 0.57 | 0.69 | 0.66 | 0.47 | 0.57 | 0.70 | 0.73 | 0.39 |
|  | BCP (lag 1) only^b^ |  | 0.67 | 0.81 | 0.79 | 0.52 | 0.64 | 0.82 | 0.82 | 0.44 |
|  | BCP (lag 1) and traffic/air pollution exposures | 0.67 | 0.66 | 0.84 | 0.89 | 0.42 | 0.64 | 0.83 | 0.88 | 0.39 |
|  | Top 10 risk factors | 0.71 | 0.71 | 0.77 | 0.77 | 0.55 | 0.69 | 0.75 | 0.74 | 0.55 |
| Non-Asthmatics (No rhinitis) | All predictors | 0.71 | 0.71 | 0.77 | 0.73 | 0.59 | 0.69 | 0.74 | 0.74 | 0.55 |
|  | Sociodemographic | 0.53 | 0.54 | 0.49 | 0.63 | 0.42 | 0.56 | 0.58 | 0.67 | 0.39 |
|  | Indoor/home exposures | 0.53 | 0.54 | 0.49 | 0.62 | 0.42 | 0.57 | 0.49 | 0.66 | 0.41 |
|  | Traffic/Air pollution exposures | 0.51 | 0.53 | 0.50 | 0.70 | 0.32 | 0.51 | 0.58 | 0.76 | 0.25 |
|  | Symptoms/medication use | 0.67 | 0.69 | 0.78 | 0.76 | 0.55 | 0.67 | 0.77 | 0.71 | 0.58 |
|  | Asthma/eczema | 0.55 | 0.57 | 0.69 | 0.63 | 0.49 | 0.57 | 0.69 | 0.72 | 0.40 |
|  | BCP (lag 1) only^b^ |  | 0.67 | 0.81 | 0.80 | 0.51 | 0.64 | 0.82 | 0.84 | 0.43 |
|  | BCP (lag 1) and traffic/air pollution exposures | 0.67 | 0.66 | 0.84 | 0.89 | 0.42 | 0.64 | 0.83 | 0.88 | 0.39 |
|  | Top 10 risk factors | 0.71 | 0.71 | 0.78 | 0.76 | 0.57 | 0.69 | 0.75 | 0.72 | 0.56 |

^a^ The accuracy, sensitivity, and specificity were calculated based on the optimal threshold that was determined by using the predicted probabilities from the cross-validation set.

^b^ Cross validation was not able to apply to the GBM models with 1 predictor variable. Thus, CV AUC and optimal number of tree based on cross validation were not produced. The total number of 2000 trees was used in the GBM models with 1 predictor variable.

Figure S1. Boxplot of relative influence, for 50 different random training sets, of the top 10 risk factors in models fit using all predictor variables for non-asthmatics, non-asthmatics (rhinitis), and non-asthmatics (no rhinitis).

Table S5. Comparison of gradient boosting models^a^ vs. logistic regression^b^ for all participants, asthmatics, and non-asthmatics averaged across 50 training sets.

| Models | Groups | AUC: Across-participant test set | AUC: Within-participant test set |
| --- | --- | --- | --- |
| Logistic regression | All participants | 0.77 | 0.74 |
|  | Asthmatics | 0.71 | 0.67 |
|  | Non-asthmatics | 0.72 | 0.67 |
| Gradient boosting model | All participants | 0.78 | 0.75 |
|  | Asthmatics | 0.71 | 0.68 |
|  | Non-asthmatics | 0.72 | 0.69 |

^a^ Gradient boosting model was fit with the top 10 predictors with the highest median relative influence across the 50 training sets, interaction depths of 1, and 5-fold cross validation.

^b^ Logistic regression model was fit with the same 10 predictors, where some of the continuous variables were categorized, across the 50 training sets.

Table S6. Logistic regression^a^ results for all participants, asthmatics, and non-asthmatics for a random training set.

| Predictors^c,d^ | All Participants | | Asthmatics | | Non-asthmatics | |
| --- | --- | --- | --- | --- | --- | --- |
|  | OR | 95% CI^b^ | OR | 95% CI^b^ | OR | 95% CI^b^ |
| Intercept | 0.09 | (0.07-0.11) | 0.20 | (0.09-0.42) | 0.07 | (0.04-0.11) |
| Age ^c^ |  |  |  |  |  |  |
| [8-14 years old] | 0.62 | (0.48-0.80) | 0.65 | (0.44-0.97) | 0.63 | (0.45-0.89) |
| [>14 years old] | 0.39 | (0.06-1.39) | 0.59 | (0.03-4.51) | 0.32 | (0.02-1.68) |
| CALINE4 freeway NOx ^d^ | 0.97 | (0.90-1.04) |  |  | 1.01 | (0.92-1.09) |
| CALINE4 non-freeway NOx ^c^ |  |  |  |  |  |  |
| [7-15 ppb] | 1.45 | (1.10-1.91) | 1.74 | (1.17-2.60) | 1.08 | (0.73-1.57) |
| [>15 ppb] | 2.51 | (1.36-4.47) | 2.19 | (0.75-6.53) | 2.26 | (1.04-4.63) |
| Any asthma medication use [Yes] | 1.04 | (0.72-1.49) |  |  |  |  |
| BMI percentile [>95] ^c^ | 1.01 | (0.73-1.38) | 0.87 | (0.54-1.40) | 1.09 | (0.69-1.68) |
| Wheeze symptom [Yes] | 1.60 | (1.13-2.26) | 1.78 | (1.18-2.69) | 1.93 | (1.12-3.28) |
| Rhinitis symptoms [Yes] | 1.70 | (1.21-2.37) |  |  | 1.91 | (1.21-2.96) |
| Itchy eye symptoms [Yes] | 1.37 | (0.98-1.91) | 1.27 | (0.86-1.89) | 1.64 | (1.02-2.65) |
| Ever asthma status [Yes] | 1.93 | (1.41-2.62) |  |  |  |  |
| BCP [Yes] | 3.48 | (2.64-4.58) | 3.01 | (2.03-4.49) | 3.95 | (2.69-5.79) |
| Education level |  |  | 0.94 | (0.79-1.12) |  |  |
| Parental stress [>7] ^c^ |  |  | 1.65 | (1.07-2.56) |  |  |
| 24-hour average: PM_2.5_^c^ |  |  |  |  |  |  |
| [10-20 µg/m^3^] |  |  | 1.21 | (0.73-2.01) |  |  |
| [>20 µg/m^3^] |  |  | 1.25 | (0.72-2.19) |  |  |
| Age of asthma onset >9 ^c^ |  |  | 1.21 | (0.73-2.00) |  |  |
| 24-hour average: NO_2_ ^c^ |  |  |  |  |  |  |
| [10-25 ppb] |  |  |  |  | 1.02 | (0.61-1.79) |
| [>25 ppb] |  |  |  |  | 1.13 | (0.63-2.09) |
| Parent history of asthma [Yes] |  |  |  |  | 1.43 | (0.96-2.09) |

^a^ Logistic regression model was fit with predictors with the highest median relative influence across the 50 training sets from the gradient boosting model. The regression model was fit on one of the random training sets.

^b^ The 95% CIs were calculated using the Wald test.

^c^ Age was categorized as 0-8, 8-14, and 14+ years old; BMI percentiles were categorized as 0-95 and 95+; non-freeway NOx was categorized as 0-7, 7-15, and 15+ ppb; age of asthma onset was categorized at 0-9 and 9+; PM_2.5_ was categorized as 0-10, 10-20 and 20+ µg/m^3^; parental stress was categorized at 0-7 and 7+; NO2 was categorized as 0-10, 10-25, and 25+ ppb.

^d^ The adjusted OR for bronchitic symptoms associated with a 10 µg/m^3^ increase in CALINE4 freeway NOx.

Figure S2. Area under the receiver operating characteristic curve (AUC) of the gradient boosting models and logistic regression model models fit separately with all risk factors and top 10 most important risk factors for 50 different random across-participant holdout test datasets.

Figure S3. Area under the receiver operating characteristic curve (AUC) of the gradient boosting models and logistic regression models fit separately with all risk factors and top 10 most important risk factors for 50 different random within-participant holdout test datasets.
